# Duality quantum algorithm efficiently simulates open quantum systems

**DOI:** 10.1038/srep30727

**Published:** 2016-07-28

**Authors:** Shi-Jie Wei, Dong Ruan, Gui-Lu Long

**Affiliations:** 1 State Key Laboratory of Low-dimensional Quantum Physics and Department of Physics, Tsinghua University, Beijing 100084, P. R. China; 2 Tsinghua National Laboratory for Information Science and Technology, Beijing 100084, P. R. China; 3 Collaborative Innovation Center of Quantum Matter, Beijing 100084, China

## Abstract

Because of inevitable coupling with the environment, nearly all practical quantum systems are open system, where the evolution is not necessarily unitary. In this paper, we propose a duality quantum algorithm for simulating Hamiltonian evolution of an open quantum system. In contrast to unitary evolution in a usual quantum computer, the evolution operator in a duality quantum computer is a linear combination of unitary operators. In this duality quantum algorithm, the time evolution of the open quantum system is realized by using Kraus operators which is naturally implemented in duality quantum computer. This duality quantum algorithm has two distinct advantages compared to existing quantum simulation algorithms with unitary evolution operations. Firstly, the query complexity of the algorithm is *O*(*d*^3^) in contrast to *O*(*d*^4^) in existing unitary simulation algorithm, where *d* is the dimension of the open quantum system. Secondly, By using a truncated Taylor series of the evolution operators, this duality quantum algorithm provides an exponential improvement in precision compared with previous unitary simulation algorithm.

Quantum computer works quantum mechanically^1^,^2^, and can efficiently factorize large numbers^3^ and search in an unsorted database^4^,^5^. Simulation of quantum systems is one of the most important original motivations of coming up with the idea of quantum computers^1^, and the progress of quantum simulation study is developing fast^6^,^7^,^8^,^9^,^10^,^11^,^12^,^13^,^14^,^15^,^16^. The dynamic evolutions of a closed system are described by unitary transform, which can be simulated in quantum computer directly. However, in the real world, quantum systems interact with their surrounding environment inevitably, hence most systems are open systems. The dynamic evolution of an open quantum system is usually non-unitary because of decoherence and dissipation. It is natural to describe the dynamics of an open quantum system by including the interaction between the principal system and an environment^6^. The principal system and the environment coupled together form a closed quantum system, which is denoted as total system. Assume that the Hilbert space of principal system is 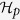 with dimensions *d*_*p*_ and the Hilbert space of environment is 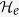 with dimensions *d*_*e*_, then the Hilbert space for the total quantum system consisting of principal system and environment is 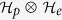
7. We assume that the system-environment state is the product state in the beginning, and the joint density matrix is described as *ρ* ⊗ *ρ*_*env*_. Considering the dynamics of the principal system is what we interested, the evolution of the density matrix after performing a partial trace over environment is^6^


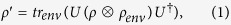


where *ρ*′ is the density matrix of the final state of principal system and *U* is time evolution operator imposed on the total system. The corresponding Hamiltonian *H* of *U* is in the space 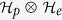. For convenience, we assume that the dimensions of principal system and environment are the same, namely, *d* = *d*_*p*_ = *d*_*e*_. The dimension of total Hamiltonian *H* is *d*^2^. Lloyd firstly proposed a quantum algorithm to simulate open quantum system efficiently^7^. In this algorithm, by enlarging the system to include the environment, the total system Hamiltonian is decomposed in the form 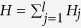 where each 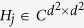 is Hermitian and satisfies 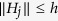 for a given constant *h*. The query complexity of simulating time evolution of the open quantum system in an accuracy *ε* over time *t* is approximated to *O*(*ld*^4^*ht*^2^/*ε*). By regarding the total system as a bigger closed quantum system, this algorithm performs unitary transformation as same as in the closed system.

The concept of duality quantum computers is first proposed by Long in 2002 based on the general principle of quantum interference^17^,^18^, which draw many attentions^18^,^19^,^20^,^21^,^22^,^23^,^24^,^25^,^26^,^27^,^28^,^29^,^30^,^31^,^32^,^33^,^34^,^35^,^36^,^37^,^38^,^39^,^40^,^41^,^42^,^43^,^44^,^45^. It is shown that any bounded linear operator can be expressed as a linear combinations of unitary operators in a duality quantum computer^21^. Thus, duality quantum computers can perform non-unitary transformation and provide novel way to design quantum algorithms, which can adapt the techniques in classical algorithm design to quantum algorithms, already showing flexibility and good performance in precision for closed quantum systems. Recently, several duality quantum algorithms have been proposed, which simulate Hamiltonian dynamics by linear combinations of unitary operations in a closed quantum system^46^,^47^,^48^,^49^. In the algorithms in refs 47, 48, 49, the performance has exponential improvement in the dependence on precision.

Alternatively, in an open quantum system coupled with surrounding environment, the dynamics can also be described by a completely positive linear map *ε*(*ρ*). The quantum operations can be represented in operator-sum representation by Kraus operators. Suppose the initial state of environment is a pure state, denoted as *ρ*_*env*_ = |*e*_0_〉 〈*e*_0_|. Equation (1) can be rewritten as^6^


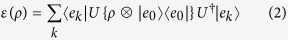



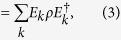


where *E*_*k*_ ≡ 〈*e*_*k*_|*U*|*e*_0_〉 is an Kraus operator and satisfies completeness relation 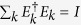. The complete set of 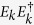 is known as a “Positive Operator-Valued Measure”. It should be noted that the operator *E*_*k*_ is only acted on the principle system. So, if we can realize the Kraus operator, the complexity of evolution simulation that is dependent on dimensions will be decreased. Generally speaking, Kraus operator *E*_*k*_ is non-unitary and can not be realized in quantum computer directly. However, the Kraus operator can be realized in a duality quantum computer^40^,^41^. In our method, the query complexity is 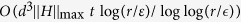, which exponentially improved the performance of quantum algorithm in ref. 7.

In this paper, we present a duality quantum algorithm to simulate Hamiltonian evolution for an open quantum system. There are two stages in our method. The first stage realizes Kraus operators in the duality quantum computer. The second stage of the algorithm is based upon a truncated Taylor series to approximate the evolution operators. The query complexity of the algorithm is significant decreased compared with Lloyd’s algorithm^7^. We demonstrate this algorithm by a single quibit open quantum system as an example.

## Results

### Realization of Kraus operators in duality quantum computer

**Figure 1:**
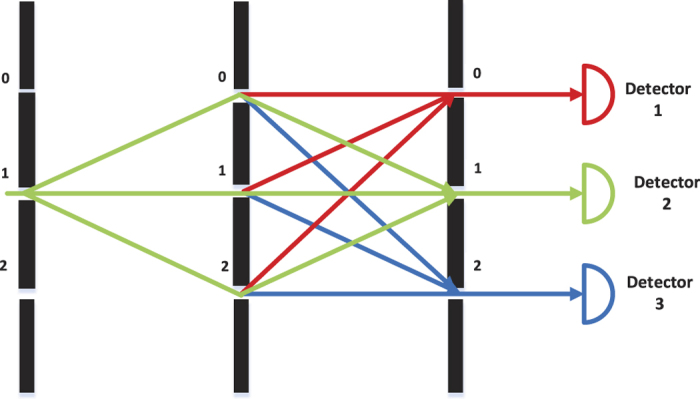
An illustrator for a three-slits duality quantum computer.

A duality quantum computer is a moving quantum computer passing through a *d*-slit which exploits the wave-particle duality of quantum systems^17^. The physic picture is : a quantum system passing through a *d*-slits with its wave function being divided into *d* sub-waves, the dividing operation denoted as the quantum wave divider (QWD) operation. Different unitary operations are performed simultaneously on the sub-waves at different slits. This is called the duality parallelism, and it enables the duality quantum computer to perform non-unitary gate operations. Conversely, the quantum wave combiner (QWC) operation adds up all the sub-waves into one wave function. Compared to ordinary quantum computers in which only unitary operators are allowed, One can perform different gate operations on the sub-wave functions at different slits in the duality quantum computer^17^. Generally, we only measure the final wave functions on 0-slit to realize a duality quantum gate, which is called single output duality quantum computing. Furthermore, we make measurements of the final wave functions on all *d*-slits, which is called complete measurements. After detecting, through QWD operation and QWC operation, every path on each-slit realized a duality quantum gate. It means that *d* duality quantum gates are performed in one process. The process is denoted as multi-output duality quantum computing. Duality quantum gates are generally non-unitary and naturally suitable to perform non-unitary evolutions. A three-slits duality quantum computer is shown in Fig. 1. The input is from the 0-th slit, and it is divided into three sub-waves by the middle screen with three slits. After the middle screen, different operations are performed on the different sub-waves, and three outputs of duality quantum computing are collected from three-slits on the right wall^18^.

It has been proven that a moving *n*-qubit duality computer passing through a *d*-slit can be perfectly simulated by an ordinary quantum computer with *n*-qubit and an extra qudit resource^18^,^19^,^20^, which is called duality quantum computing mode. For the convenience, we use the expressions from duality quantum computing mode^19^,^20^,^32^,^33^ in this article.

**Figure 2:**
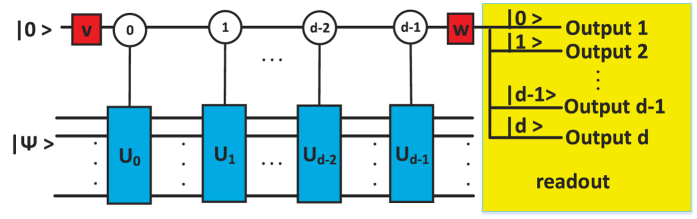
The multi-output duality quantum computing circuit in a quantum computer. |Ψ〉 denotes the initial state of work qubit, and |0〉 is the initial state of the controlling auxiliary qudit. The circles represent the state of the controlling qudit and the squares represent unitary operations. Unitary operations *U*_0_, *U*_1_ 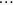, *U*_*d*−1_ are activated only when the qudit holds the respective values indicated in circles^18^. The “readout” part marked by yellow rectangle means that: When the auxiliary qudit in the |*j*〉 state, where *j* ∈ {0, 1 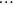, *d*}, the corresponding output (final state) of the work qubit will be redout by corresponding detector.

The *n*-qubit ordinary quantum computer is represented by *n* work qubit and an auxiliary qudit represents a *d*-slits. The QWD operation can be represented by a general unitary operation *V* and the QWC operation can be represented by a general unitary operation *W*. The two unitary operations act on an auxiliary qudit. There are *d* controlled unitary operations act on ordinary quantum computer between the operations *V* and *W*. The quantum circuit of duality quantum computer is given in Fig. 2.

It is convenient to divide the whole process into four steps to illustrate the multi-output duality computing in a quantum computer.

#### Step one

The quantum system is prepared with initial state |Ψ〉|0〉 firstly. The QWD operation is implemented by performing the operator *V* on the auxiliary qudit |0〉, and this operation transforms the initial state into


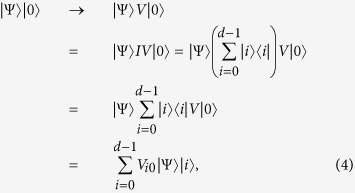


where *V*_*i*0_ = *p*_*i*_ is a complex number and satisfies the condition 
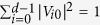
, |*V*_*i*0_| ≤ 1. *V*_*i*0_ represents the divider structure and is the first column element of the unitary matrix *V* representing the coefficient in each slit. The closure condition ∑ |*i*〉〈*i*| = I in quantum mechanics has been used in the deviation. The final state |Ψ〉|*i*〉 represents the sub-wave at the *i*-th slit.

#### Step two

Performing the auxiliary qudit controlled operations *U*_0_, *U*_1_ 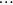, *U*_*d*−1_ on the work qubits with initial state |Ψ〉 which leads to the following transformation,


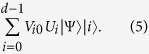


The corresponding physical picture is that unitary operations are implemented simultaneously on the sub-waves at different slits.

#### Step three

Performing the unitary operation *W* on the auxiliary qudit |*i*〉. Then the following state is obtained,


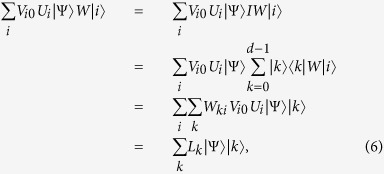


where *L*_*k*_ = ∑_*i*_*W*_*ki*_*V*_*i*0_*U*_*i*_ is the duality quantum gate. In previous paper^17^,^18^, only *L*_0_ is studied as a duality quantum gate. In this article, we discuss all the *k* number duality quantum gates.

#### Step four

After step three, the auxiliary qudit is in a superposition state. Making the complete measurements, namely, measuring the final wave function when the qudit is in state |*j*〉 by placing *j* detectors at *j* different slits. which described as “readout” in Fig. 2. The complete measurements are also clearly visualized by the detectors in Fig. 1.

The duality quantum gate, or generalized quantum gate is defined as follows


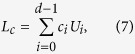


where *U*_*i*_ is unitary and *c*_*i*_ is the complex coefficient and satisfies


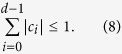


When *c*_*i*_ is restricted to positive real, *c*_*i*_ is denoted by *r*_*i*_, and satisfies the constrained condition of ∑_*i*_*r*_*i*_ ≤ 1. In this scenario, the duality quantum gate is called real duality gate which is denoted as *L*_*r*_. So, the form of real duality quantum gate can be expressed as


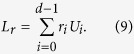


This corresponds to a physical picture of an asymmetric *d*-slit, and *r*_*i*_ is the probability that the duality computer system passes through the *i*-th slit.

Because unitary operators have the unclosed property under addition, the duality quantum gates are generally non-unitary. Moreover, Gudder has proved that all linear bounded operators in a finite dimensional Hilbert space can be expressed as an element in the positive cone of generalized quantum gates^21^. Many recent studies about the mathematical theory of duality quantum computer have been made^19^,^20^,^21^,^22^,^23^,^24^,^25^,^26^,^27^,^28^,^29^,^30^,^31^,^34^,^36^.

**Theorem.** The duality quantum gate *L*_*k*_ = ∑_*i*_*W*_*ki*_*V*_*i*0_*U*_*i*_ is a trace preserving Kraus operator, namely, 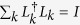.

Proof. Defining *L*_*k*_ = ∑_*i*_*W*_*ki*_*V*_*i*0_*U*_*i*_ firstly, then we have, 
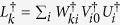
. Then a straightforward derivation gives


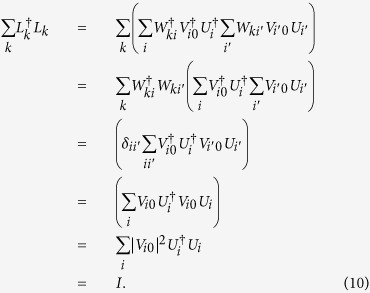


The conditions that matrices *W, V* and operator *U*_*i*_ are unitary are used in the proof. So, the duality quantum gate *L*_*k*_ can be applied to realize Kraus operator *E*_*k*_. Actually, *E*_*k*_ is a bounded linear operator in a finite dimensional Hilbert space which can be decomposed into a sum of unitary operators. By extending the Hilbert space, any Kraus operator *E*_*k*_ can be realized by duality quantum gate *L*_*k*_. The effect of environment on the principal system can be explained as the combination effects of different operators performed on the system. In the expression of completely positive linear map form, we can get the dynamic evolution result of the open system directly without coupled with environment.

### Simulating the time evolution of open quantum system

We have realized the Kraus operator *E*_*k*_ in the previous section. To perform the whole duality quantum algorithm, we only need to realize the unitary operator *U*_*i*_ in the Kraus operator in next step. *U*_*i*_ in Eq. 6 is regarded as a time evolution operator and it is approximated by a truncated Taylor series. It can be realized in a duality quantum computer just as in the BCCKS algorithm^35^,^48^.

In the algorithm, consider a quantum system with Hamiltonian 
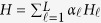
 where each 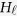 is unitary. Dividing the finite length evolution time *t* into *n* segments, with each segment of length *t*/*n*. The time evolution operator of each segment is approximated as


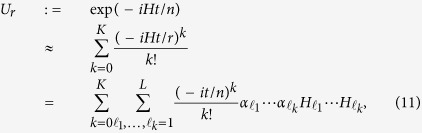


where *K* is the order of Taylor series.

Without loss of generality, let each 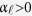. The approximation 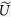 is a linear combinations of unitary operations because of the assumption that 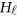 is unitary. It leads to the approximated expression of *U*_*r*_ has a quantum duality gate form. The truncated Taylor series index is denoted as^48^


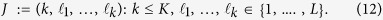


Then, the expression of 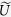 can be simplified as


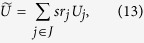


where 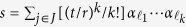, 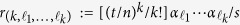 and 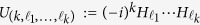.

According to Eq. (10), 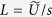 is a quantum duality gate and *s* is the normalization constant.

**Figure 3:**
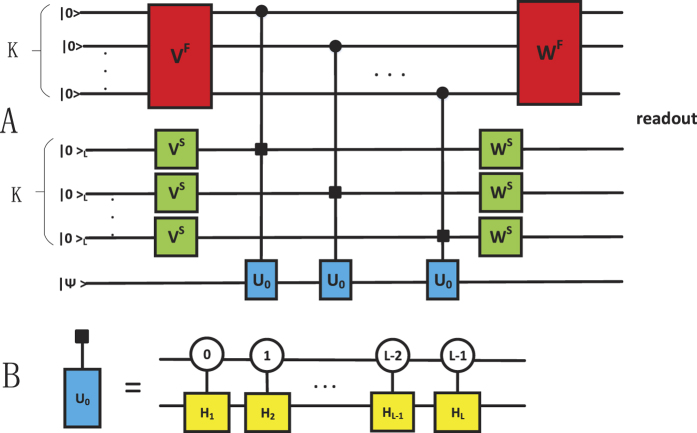
Quantum circuit for the BCCKS algorithm in single output duality quantum computing. In Part *A* of Fig. 3, |Ψ〉 is the initial state of work qubit and there are *K* numbers of |0〉 auxiliary controlling qubits and *K*numbers of *L* level |0〉_*L*_ auxiliary controlling qudits in the auxiliary system. *K* numbers of |0〉 auxiliary controlling qubits control the *K*numbers of *L* level |0〉_*L*_ auxiliary controlling qudits and the unitary operations *U*_0_ are activated only when the *L* level |0〉_*L*_ auxiliary controlling qudits hold the respective values indicated in circles. Part *B* of Fig. 3 is to illustrate that each unitary operation *U*_0_ is composed of *H*_1_, *H*_2_, …, *H*_*L*−1_, *H*_*L*_. We only “readout” the result with the auxiliary system in state 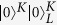.

We give the quantum circuit for realizing the approximation 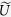 is given in Fig. 3, which is the same as that in ref. 35. The part *A* of Fig. 3 is the implementation of 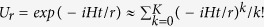. The controlled unitary operation *U*_0_ which illustrated in part *B* corresponds to the linear combination form of the Hamiltonians, 
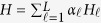
. The quantum circuit realizes the evolution in a segment,


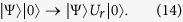


The implementation of operation 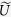 needs an auxiliary system and a work system(target state). The auxiliary system is composed by *K* auxiliary qubits |0〉^*K*^ and *K* numbers of *L* level auxiliary qudits |0〉_*L*_, for the implementations of two QWD operations and two QWC operations. We denote the initial state of the whole system as 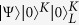, where |Ψ〉 is the work qubit state and 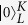 means *K* numbers of *L* level auxiliary qudits all in state |0〉_*L*_^35^.

The first QWD can be expressed as a 2^*K*^ × 2^*K*^ matrix, denoted as *V*^*F*^. Defining 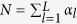, the elements of the matrix is


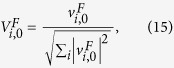


where


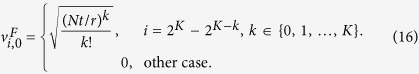


Similarly, we denote the second QWD operation as *V*^*S*^, which can be viewed as a *L* × *L* matrix. The elements of the matrix satisfy


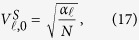


Corresponding to *K* auxiliary qubits |0〉^*K*^, *K* numbers of *L* level |0〉_*L*_ auxiliary controlling qudits should be transformed into *K* numbers of state 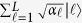 by the same QWD operation *V*^*S*^. They can be denoted as


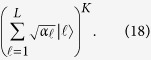


Applying the two QWD operations *V*^*F*^and *V*^*S*^ to the state 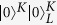 produces the state of total auxiliary system


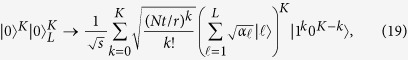


where *s* = ∑_*j*∈*J*_*β*_*j*_ is the normalization constant. We perform the auxiliary system controlled operation *U*_*j*_ on the work system. The state of the whole system is transformed into


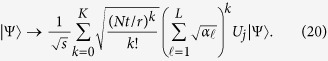


Then, we need to perform two QWC operations to combine the wave functions, denoted as *W*^*F*^ = (*V*^*F*^)^†^ and *W*^*S*^ = (*V*^*S*^)^†^, respectively. Physically, the two QWC operations are the counterparts of the two QWD operations.

We denote the state orthogonal to 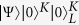 as |Φ〉, the total process can be described as:


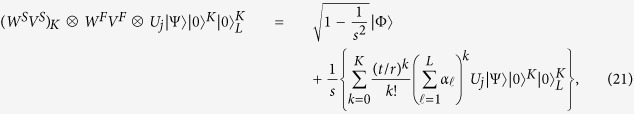


where(*W*^*S*^*V*^*S*^)_*K*_ means *K* numbers of *W*^*S*^*V*^*S*^ operations and *U*_*j*_ corresponds to some 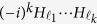 and *j* ∈ *J*.

The results of the duality quantum computing are in the terms with the auxiliary system in state 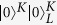. Therefore, we only need to readout the output of the work system with auxiliary system in state 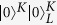, which corresponds to the single-output duality quantum computing. Namely, the initial state goes through the transformation we interested is


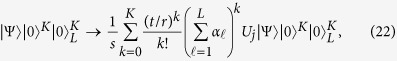


Thus, we obtain


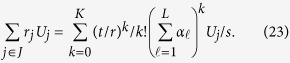


Consequently, we have successfully realized the following process,


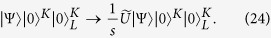


If we make measurement directly, the probability of detecting the auxiliary state 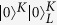 is *P*_*s*_, where 
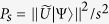
. Namely the probability of implementing *U* on the target state |Ψ〉 successfully is *P*_*s*_. Amplifying the amplitude of the desired term before the measurement by applying the robust obvious amplitude amplification given in Res.^48^ enables us to nearly deterministically implement 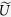. The accuracy of approximation of 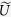 can be quantified by approximation error 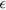. Consider the case that all 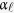 equal to *m* corresponding Hamiltonian is approximately decomposed into equal-sized parts. To ensure the total error of simulating time evolution under 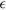, *m* should be in the order 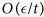. The terms of Hamiltonian decomposition are 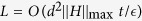. The number of segments is in the order 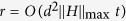. According to the Chernoff bound^47^, the query complexity in each segment is


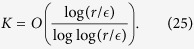


The query complexity for the full simulation algorithm is *r* times *K*. Consider all the operations performed on auxiliary system and work system, the total number of gates in the simulation for time *t*/*r* in each segment is^48^


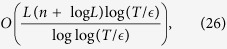


where *T* = (*α*_1_ + 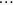 + *α*_*L*_)*t*.

In last section, we have realized Kraus operator by the duality quantum gate *E*_*k*_ = *L*_*k*_ = ∑_*i*_*W*_*ki*_*V*_*i*0_*U*_*i*_. In this section, unitary operator *U*_*i*_ is realised by BCCKS algorithm in duality quantum computing form with precision 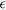. So, we have successfully simulated the total evolution of an open quantum system,


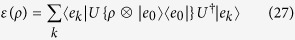



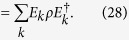


The complexity of performing *U*_*i*_ with precision 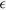 is *Kr*. Consider the fact that the coefficients satisfy ∑_*i*_|*W*_*ki*_*V*_*i*0_| ≤ 1, the complexity of performing *E*_*k*_ is as the same as the complexity of performing *U*_*i*_. The total complexity of the whole algorithm with *d* numbers of *E*_*k*_ is


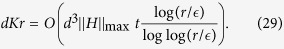


Compared with the complexity of Llyod ‘s algorithm *O*(*ld*^4^*ht*^2^/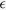), the dependence on dimension of principal system is decreased from *O*(*d*^4^) to *O*(*d*^3^) and the performance is exponential improved on precision 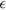. An example to show the implementation of this simulation algorithm is given in next section.

### Application to a single quibit open quantum system

Suppose we have a principal system with single qubit, interacting with a single qubit environment. *U* is time evolution operator imposed on the total system^6^. The expression of *U* is


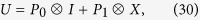


where *X* represents the usual Pauli matrix acting on the environment, and *P*_0_ = |0〉〈0|, *P*_1_ = |1〉〈1| are projectors acting on system. The initial state of environment is |0〉. In this special case, the number of state *k* is 2. Equation (1) is simplified to


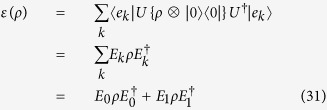


where *E*_0_ = *P*_0_, *E*_1_ = *P*_1_, and satisfies completeness relation 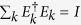. *E*_0_ and *E*_1_ can be realised by duality quantum gate *L*_0_ = ∑_*i*_*W*_0*i*_*V*_*i*0_*U*_*i*_ and *L*_1_ = ∑_*i*_*W*_1*i*_*V*_*i*0_*U*_*i*_ respectively. Assume that


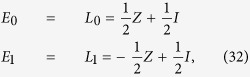


where *Z* is the usual Pauli matrix and *U*_0_ = *Z, U*_1_ = *I*. So, the QWD operator *V* and the QWC operator *W* are chosen as


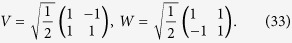


**Figure 4:**
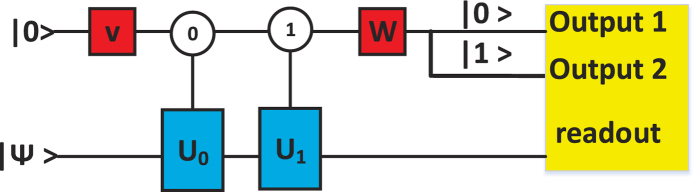
Quantum circuit of realisation of Kraus operator in duality quantum computing when *d* = 2. |Ψ〉 denotes the initial state of principal system, and environment is in the |0〉 state. The squares represent unitary operations and the circles represent the state of the controlling qubit. Unitary operations *U*_0_, *U*_1_ are activated only when the auxiliary qubit is |0〉 and |1〉 respectively.

Measuring the final wave functions when the qudit is in state |0〉 and |1〉 by placing two detectors as shown by “readout” in Fig. 4. We have realized the trace preserving Kraus operator *E*_*k*_. Then, regarding *U*_*i*_ as a time evolution operator, it can be realized by BCCKS algorithm in a duality quantum computer. Ignoring the global phase factor, *U*_0_ can be regarded as 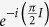. Similarly, *U*_1_ can be expressed as 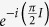. Regarding the evolution time as *t* = 1, the corresponding Hamiltonian of *U*_0_ and *U*_1_ are *H*^0^ and *H*^1^. They can be expressed as


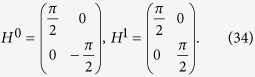


After obtaining the expression of *U*_0_ and *U*_1_ and finding the corresponding Hamiltonian, we are able to simulate the Hamiltonian by approximating the truncated Taylor series of the evolution operator in duality quantum computer. The process of realizing *U*_0_ or *U*_1_ has given in the last section.

## Discussion

In the present paper, we have briefly described the dynamics of an open quantum system and the quantum operations can be elegantly represented in operator-sum representation. The dynamics in the principal system can be described by trace preserving Kraus operators. The duality quantum computing is a suitable way to realise Kraus operators with non-unitary feature. Duality quantum computer provides the capability to perform linear combinations of unitary operations in the computation, which is called the duality quantum gates or the generalized quantum gates. The duality quantum computer can be perfectly simulated by an ordinary quantum computer with *n*-qubit and an additional qudit resource. By realizing Kraus operators through duality quantum computing, and approximating Hamiltonian simulation by the truncated Taylor series of the evolution operator in duality quantum computer, we present an efficient quantum algorithm for simulating Hamiltonian in open quantum system. Consider the fact that all quantum system is inevitable coupled with its environment in the real world, our method can be applied in a class of general physical systems. By realizing Kraus operators, the query complexity is decrease from *O*(*d*^4^) dimension dependence to *O*(*d*^3^) of the open quantum system. Moreover, through the use of truncated Taylor series in duality computing, our algorithm can provide an exponential improvement in precision.

## Additional Information

**How to cite this article**: Wei, S.-J. *et al*. Duality quantum algorithm efficiently simulates open quantum systems. *Sci. Rep.* **6**, 30727; doi: 10.1038/srep30727 (2016).

## References

[b1] Feynman R. P. Simulating physics with computers. Int. J. Theor. Phys. 21, 467 (1982).

[b2] Benioff P. The computer as a physical system: A microscopic quantum mechanical Hamiltonian model of computers as represented by Turing machines. J. Stat. Phys. 22, 563–591 (1980).

[b3] Shor P. W. Algorithms for quantum computation: Discrete logarithms and factoring. Foundations of Computer Science, 1994 Proceedings, 35th Annual Symposium on. IEEE, 124–134 (1994).

[b4] Grover L. K. A fast quantum mechanical algorithm for database search. Proceedings of the twenty-eighth annual ACM symposium on Theory of computing. ACM, 212–219 (1996).

[b5] Long G. L. Grover algorithm with zero theoretical failure rate[J]. Phys. Rev. A 64, 022307 (2001).

[b6] Nielsen M. A. & Chuang I. L. Quantum Computation and Quantum Information (Cambridge University Press, 2000).

[b7] Lloyd S. Universal quantum simulators. Science. 273, 1073 (1996). 8688088 10.1126/science.273.5278.1073

[b8] Gerritsma R. . Quantum simulation of the Dirac equation. Nature 463, 68–71 (2010). 20054392 10.1038/nature08688

[b9] Feng G. R., Xu G. F. & Long G. L. Experimental realization of nonadiabatic holonomic quantum computation. Phys Rev Lett. 110, 190501 (2013). 23705695 10.1103/PhysRevLett.110.190501

[b10] Kim K. . Quantum simulation of frustrated Ising spins with trapped ions. Nature, 465, 590–593 (2010). 20520708 10.1038/nature09071

[b11] Feng G. R. . Experimental simulation of quantum tunneling in small systems. Sci Rep 3, 2232 (2013). 23958996 10.1038/srep02232 PMC3747511

[b12] Lanyon B. P. . Universal digital quantum simulation with trapped ions. Science 334(6052), 57–61 (2011). 21885735 10.1126/science.1208001

[b13] Lu Y. . Experimental digital quantum simulation of temporal-spatial dynamics of interacting fermion system. Sci. Bull 2, 241–248 (2015).

[b14] Sornborger A. T. Quantum simulation of tunneling in small systems. Sci Rep 2, 597 (2012). 22916333 10.1038/srep00597 PMC3424524

[b15] Zhang C., Li C. F. & Guo G. C. Experimental demonstration of photonic quantum ratchet. Sci. Bull 2, 249–255 (2015).

[b16] Jin F. Z. . Experimental simulation of the Unruh effect on an NMR quantum simulator[J]. Sci China-Phys Mech Astron 3, 630302 (2016).

[b17] Long G. L. General quantum interference principle and duality computer. Commun. Theor. Phys. 45, 825–844 (2006).

[b18] Long G. L. Duality quantum computing and duality quantum information processing. Int. J. Theor. Phys. 50, 1305–1318 (2011).

[b19] Long G. L. & Liu Y. Duality computing in quantum computers. Commun. Theor. Phys. 50, 1303–1306 (2008).

[b20] Long G. L., Liu Y. & Wang C. Allowable generalized quantum gates. Commun. Theor. Phys. 51, 65–67 (2009).

[b21] Gudder S. Mathematical theory of duality quantum computers. Quantum Inf. Process. 6, 37–48 (2007).

[b22] Long G. L. Mathematical theory of the duality computer in the density matrix formalism. Quantum. Inf. Process. 6(1), 49–54 (2007).

[b23] Gudder S. Duality quantum computers and quantum operations. Int. J. Theor. Phys. 47, 268–279 (2008).

[b24] Wang Y. Q., Du H. K. & Dou Y. N. Note on generalized quantum gates and quantum operations. Int. J. Theor. Phys. 47, 2268–2278 (2008).

[b25] Du H. K., Wang Y. Q. & Xu J. L. Applications of the generalized LÃŒders theorem. J. Math. Phys. 49, 013507 (2008).

[b26] Cao H. X., Li L., Chen Z. L., Zhang Y. & Guo Z. H. Restricted allowable generalized quantum gates. Chin. Sci. Bull. 55, 2122–2125 (2010).

[b27] Zhang Y., Cao H. X. & Li L. Realization of allowable qeneralized quantum gates. Sci China-Phys Mech Astron 53, 1878–1883 (2010).

[b28] Chen L., Cao H. X. & Meng H. X. Generalized duality quantum computers acting on mixed states. Quantum Information Processing 11, 4351–4360 (2015).

[b29] Cao H. X., Chen Z. L., Guo Z. H. . Complex duality quantum computers acting on pure and mixed states. Sci. China-Phys Mech Astron 55, 2452–2462 (2012).

[b30] Cao H. X. . Mathematical theory of generalized duality quantum computers acting on vector-states. Int. J. Theor. Phys. 52, 1751–1767 (2013).

[b31] Cui J. X., Zhou T. & Long G. L. Density matrix formalism of duality quantum computer and the solution of zero-wave-function paradox. Quantum Inf. Process. 11, 317–323 (2012).

[b32] Long G. L. & Liu Y. Duality quantum computing. Front. Comput. Sci. 2, 167–178 (2008).

[b33] Long G. L. & Liu Y. General principle of quantum interference and the duality quantum computer. Rep.Prog.Phys. 28, 410–431 (2008).

[b34] Zou X. F., Qiu D. W., Wu L. H., Li L. J. & Li L. Z. On mathematical theory of the duality computers. Quantum Inf. Process. 8, 37–50 (2009).

[b35] Wei Sh. J. & Long G. L. Duality quantum computer and the efficient quantum simulations. Quantum Information Processing. 15, **3**, 1189–1212 (2016).

[b36] Chen Z. L. & Cao H. X. A note on the extreme points of positive quantum operations. Int. J. Theor. Phys. 48, 1669–1671 (2010).

[b37] Hao L., Liu D. & Long G. L. An N/4 fixed-point duality quantum search algorithm. Sci China-Phys Mech Astron 53, 1765–1768 (2010).

[b38] Liu Y. Deleting a marked state in quantum database in a duality computing mode. Chin. Sci. Bull. 58, 2927–2931 (2013).

[b39] Hao L., Liu D. & Long G. L. An N4 fixed-point duality quantum search algorithm. Sci. China-Phys Mech Astron, 53, 1765–1768 (2010).

[b40] Cui J. X., Zhou T. & Long G. L. An optimal expression of a Kraus operator as a linear combination of unitary matrices. J. Phys. A: Math. Theo. 45 444011 (2012).

[b41] Liu Y. & Cui J. X. Realization of Kraus operators and POVM measurements using a duality quantum computer. Chin. Sci. Bull. 59, 2298–2301 (2014).

[b42] Li C. Y. & Li J. L. Allowable generalized quantum gates using nonlinear quantum optics. Commun. Theor. Phys. 53, 75–77 (2010).

[b43] Wu Z. Q., Zhang S. F. & Zhu C. X. Remarks on generalized quantum gates. Hacettepe J Math Stat, 43, 451–460 (2014).

[b44] Chen. L., Cao H. X. & Meng H. X. Generalized duality quantum computers acting on mixed states. Quantum Inf. Process. 11, 4351–4360 (2015).

[b45] Hao L. & Long G. L. Experimental implementation of a fixed-point duality quantum search algorithm in the nuclear magnetic resonance quantum system. Sci China-Phys Mech Astron. 54, 936–941 (2011).

[b46] Childs A. M. & Wiebe N. Hamiltonian simulation using linear combinations of unitary operations. Quantum. Inform. Comput. 12(**11–12**), 901–924 (2012).

[b47] Berry D. W., Childs A. M., Cleve R., Kothari R. & Somma R. D. In Proceedings of the 46th Annual ACM Symposium on Theory of Computing, New York, 2014 (ACM Press, New York, pp. 283–292 (2014).

[b48] Berry D. W., Childs A. M., Cleve R., Kothari R. & Somma R. D. Simulating Hamiltonian Dynamics with a Truncated Taylor Series. Phys. Rev. Lett. 114, 090502 (2015). 25793789 10.1103/PhysRevLett.114.090502

[b49] Babbush R. . Exponentially more precise quantum simulation of fermions in second quantization. New Journal of Physics. 18, 033032 (2016).

